# Disease progression & treatment need in sub-genotype C4 hepatitis B infection: a retrospective cohort study in the Northern Territory, Australia

**DOI:** 10.1186/s12879-025-11213-w

**Published:** 2025-07-01

**Authors:** Genevieve E. Martin, Kelly Hosking, Kelly Banz, Catherine Gargan, Geoff Stewart, Belinda Greenwood-Smith, Penelope Ramsay, Jaclyn Tate-Baker, Christine Connors, Paula Binks, Melita McKinnon, Prashanti Manchikanti, George Garambaka Gurruwiwi, Nicole Allard, Ashleigh Qama, Jessica Michaels, Emily Vintour-Cesar, Robert Batey, Catherine Marshall, Peter Nihill, Tammy-Allyn Fernandes, Karen Fuller, Steven Y. C. Tong, David Boettiger, Benjamin Cowie, Joshua S. Davis, Sarah Mariyalawuy Bukulatjpi, Jane Davies, on behalf of the Hep B PAST Partnership, Anna Ralph, Adrian Miller, Roslyn Dhurrkay, Manoji Gunthilake, Vicki Krause, Lou Sanderson, Phillip Merrdi Wilson, Genevieve Dally, Kerrie Jordan, John Boffa, Alexis Apostolellis, Amanda Dhagapan, Anna Deng, Anngie Everitt, Barbara De Graaff, Brianna Summers, Carrie Fowler, Catherine Blacker, Catherine Stoddart, Charles Pain, Cheryl Ross, David McGuinness, David Reeve, Diane Hampton, Eddie Mulholland, Ella Meumann, Elizabeth Coombes, Emma Childs, Hayden Jose, Hilary Bloomfield, Hugh Heggie, Isabelle Purcell, Jayne Porter, Jyoti Jadeja, Katherine McNamara, Katie McGuire, Keith Forrest, Kelly-Anne Stuart-Carter, Khim Tan, Leanne O’Connor, Lesley Scott, Letisha Murray, Levinia Crooks^, Linda Bunn, Lorraine Johns, Lucie Perrisel, Marco Briceno, Margaret Littlejohn, Maria Scarlett, Marilou Capati, Matthew Maddison, Mikaela Mobsby, Molly Shorthouse, Natasha Tatipata, Nicole Romero, Peter Markey, Phoebe Schroder, Rebecca Katiforis, Robyn Liddle, Rosalind Webby, Richard Sullivan, Sami Stewart, Sandra Nelson, Sean Heffernan, Sean Taylor, Shiraline Wurrawilya, Sinon Cooney, Sonja Hill, Stephen Locarnini, Steven Skov, Su Govindasamy, Sudharsan Venkatesan, Tanya Plavins, Teresa De Santis, Terese Ngurruwuthun, Tiana Alley, Timothy Nabegeyo, Vanessa Towell, Wendy Page

**Affiliations:** 1 Global and Tropical Health Division, Menzies School of Health Research, Charles Darwin University, Darwin, NT Australia; 2 Northern Territory Health, Northern Territory, Australia; 3 Department of Infectious Diseases, The University of Melbourne at the Peter Doherty Institute for Infection and Immunity, Melbourne, Australia; 4 Miwatj Health Aboriginal Corporation, Nhulunbuy, East Arnhem Land, Northern Territory Australia; 5 WHO Collaborating Centre for Viral Hepatitis, Peter Doherty Institute for Infection and Immunity, Melbourne, VIC Australia; 6 Australasian Society for HIV, Viral Hepatitis and Sexual Health Medicine (ASHM), Sydney, NSW Australia; 7 Katherine West Health Board, Katherine, Northern Territory Australia; 8 Victorian Infectious Diseases Service, The Royal Melbourne Hospital, at the Peter Doherty Institute for Infection and Immunity, Melbourne, Australia; 9 Kirby Institute, UNSW, Sydney, NSW Australia; 10 School of Medicine and Public Health, University of Newcastle, New South Wales, Australia

**Keywords:** Chronic hepatitis B, Liver cirrhosis, First Nations health, Hepatitis B virus, Viral genotype

## Abstract

**Background:**

In the Northern Territory (NT) of Australia, First Nations people with chronic hepatitis B (CHB) are infected with a unique sub-genotype, C4, which contains mutations linked to progressive fibrosis and hepatocellular carcinoma. This cohort study aimed to investigate disease progression in C4 sub-genotype infection and estimate how many untreated individuals may benefit from antiviral therapy with broadening treatment indications.

**Methods:**

Included individuals were part of Hep B PAST, a co-designed program to improve the cascade of care for people living with CHB in the NT. Disease phase and cirrhotic status were determined algorithmically using clinical and laboratory data at two time points. Loss of HBV antigens was assessed longitudinally. Treatment need was assessed cross-sectionally in the cohort at study completion. Key outcomes were estimated rates of HBsAg/HBeAg loss in sub-genotype C4 infection and quantification of how many untreated individuals qualify for therapy under current Australian and expanded global treatment guidelines.

**Results:**

HBsAg and HBeAg loss occurred at a rate of 1·04 and 8·06 events/100 person-years respectively (7342·6 and 545·6 years follow up). 783 people living with CHB were included (40% female, median age 48 years). Of these, 16% had cirrhosis (an additional 6% having FibroScan > 7 kPa, meaning 22% had cirrhosis or significant fibrosis) and 25% were prescribed antivirals. Only 6·7% of untreated individuals were treatment eligible under current guidelines. Using the 2024 World Health Organisation guidelines, this increased to 50% due mostly to fibrosis and population prevalence of diabetes.

**Conclusions:**

Despite advanced liver disease in people living with CHB in the NT, rates of antigen loss in sub-genotype C4 hepatitis B infection are similar to other genotypes. Further work is needed to understand drivers of cirrhosis and significant fibrosis in this population.

**Supplementary Information:**

The online version contains supplementary material available at 10.1186/s12879-025-11213-w.

## Background

Infection with hepatitis B virus (HBV) has a dynamic natural history reflecting interplay between the virus and host immunity. Antiviral therapy with nucleos(t)ide analogues suppresses replication of the virus, reducing risk of progression to cirrhosis and hepatocellular carcinoma (HCC) [1]. Until recently, treatment for chronic hepatitis B (CHB) has only been indicated when detectable viral replication occurs in the setting of significant hepatitis or cirrhosis [2–4]. Assessment of treatment need is complex, often requiring specialised tests and input from providers with additional training. Suggestions that broadening treatment eligibility could improve outcomes [5] and a focus on feasibility have led to a movement globally to simplify treatment algorithms and expand access to antiviral therapy, as reflected in the 2024 World Health Organisation (WHO) guidelines [6]. Whilst treatment guidelines have not typically included consideration of viral genotype, genotype is an important driver of clinical outcomes [7, 8]. For example, genotype C is associated with increased risk of HCC development [9].

In the Northern Territory (NT) of Australia a sub-genotype of hepatitis B (C4) has been uniquely described amongst First Nations people [10], a group disproportionately impacted by this infection [11, 12] and by the development of HCC [13, 14]. Seroprevalence of CHB amongst First Nations adults in the NT is 6% [11]. Sequence analysis of the C4 virus reveals frequent mutations associated in other genotypes with progressive liver disease or HCC development [15]. Among individuals with sub-genotype C4 infection, the presence of these mutations is associated with cirrhosis [16]. An initial study following 193 individuals living with sub-genotype C4 infection showed labile disease, with transition between disease phases (including onto treatment) in 9·6% of individuals per year [16]. Taken together, these early findings suggested an aggressive phenotype of sub-genotype C4 infection.

Hep B PAST (Partnership Approach to Sustainably eliminating Chronic Hepatitis B in the Northern Territory) is a participatory action research program aimed at improving care for First Nations individuals living with CHB. At completion, Hep B PAST has systematically recorded the hepatitis B serology and vaccination status of greater than 40,000 people [17]. Health services involved in the program are now exceeding national elimination strategy targets [17, 18].

Here, we used information collected through the Hep B PAST program to study clinical features of sub-genotype C4 infection. Specifically, we aimed to (a) estimate rates of HBsAg/HBeAg loss (b) quantify how many untreated individuals qualify for therapy (the “hepatitis B treatment gap”) and (c) assess the impact of expanding treatment guidelines on treatment eligibility of individuals living with sub-genotype C4 infection.

## Patients and methods

Hep B PAST is a co-designed program utilising participatory action research methodology, and includes most remote community clinics in the NT. The details of multimodal methodology of this program have been previously reported [17]. In brief, the program aimed to systematically document the HBV status of all individuals at participating clinics and link each person living with CHB to an HBV care plan. Embedding hepatitis B management in primary healthcare (providing care on country) and building local workforce capacity is an important part of this program. The provision of care for hepatitis B is performed by remote clinic staff supported by visiting multidisciplinary teams and the development and delivery of educational programs for First Nations health workforce, general practitioners and nurse practitioners.

### Participant identification

This retrospective cohort study was conducted as part of Hep B PAST. Individuals for inclusion in this study were identified using the Hep B PAST HBV status, an approach that aimed to minimise selection bias as all individuals from a given community clinic have had a status assigned. Study size was pragmatic as this study aimed to include all individuals with CHB in this setting. To estimate loss of antigens (HBsAg and HBeAg), individuals who had an HBV status indicating CHB at any time point were assessed for inclusion. For inclusion, individuals needed at least one positive HBsAg (or initial HBeAg positive), with another antigen test > 6 months afterwards. For subsequent analyses, individuals with CHB were identified using the most recent HBV status recorded in Hep B PAST. To ensure that individuals had not cleared hepatitis B since this was assigned, individuals whose most recent HBsAg was negative were reviewed and excluded if they did not have CHB.

### Clinical data

Patient demographic, clinical and laboratory data were collected as part of routine care during Hep B PAST and extracted from the Hep B Hub and participating partner clinics electronic health record (EHR) systems. Further details for key variables are described in Supplementary Methods. These data were stored in Microsoft Access (Microsoft Corporation) and compiled using STATA v17·0 (StataCorp) and R (v4·3·2). Deterministic linkage was performed using a unique identifier, the Hospital Reference Number (HRN), which is used across health services in the NT. In this setting, data linkage via HRN has been shown to be highly accurate [19] and has been used in several large population studies [20, 21].

### Inference of viral sub-genotype

Viral genotyping was not performed for all individuals involved in this study but were assumed to be sub-genotype C4 on the basis of current epidemiological knowledge. Phylogenetic analyses suggest an origin of sub-genotype C4 coincident with ancient human migration into Australia, more than 59 thousand years ago [22]. The genomes of First Nations people in northern Australia have been reported to have strong population structure, reflecting historic divergence of these communities [23]. To date, all HBV sequences from First Nations people in the NT have been sub-genotype C4 [10, 16][unpublished data]. Based on these observations, we believe it is reasonable to infer all individuals in this study are infected with sub-genotype C4 HBV.

### Data analysis

Two analysis time points (October 2020, termed baseline, and March 2024, study completion) were chosen to align with the primary analysis of Hep B PAST as this is when information about treatment status was available. The most recent value for each clinical and laboratory variable was selected in a three-year window prior to these times as follows: 1 st October 2017–30th September 2020, 1 st October 2020–20th March 2024. This window was chosen as part of a pragmatic approach to data inclusion. Values prior to the three-year window were included only in scenarios where it would be clinically appropriate for these to not be repeated (listed in Supplementary Methods).

Individuals were allocated a disease phase based on Gastroenterological Society of Australia (GESA) guidelines (phases I-IV; formerly immune tolerance, immune clearance, immune control, immune escape and occult hepatitis B), as described in Supplementary Methods and Supplementary Fig. 1 [2]. Among untreated individuals, the need for treatment was assessed against GESA guidelines [2], European Association for the Study of the Liver (EASL) guidelines [4] and 2024 World Health Organisation guidelines (as shown in Supplementary Figs. 2–4) [6]. For all guidelines, the sex-specific upper limit of normal for alanine aminotransferase (ALT) was considered 19 IU/L for females and 30 IU/L for males. For application of GESA and EASL guidelines, an individual was considered to have a detectable HBV viral load (HBV DNA) if any measurement in the three-year window prior to assessment was detectable.

Rates of antigen loss were assessed using Kaplan–Meier estimates and expressed as the number of events divided by the total number of person-years at risk. Loss of antigen was defined as occurring on the date of the first negative antigen where this was followed by a second negative value (two consecutive negative antigens), or if the final value was negative. Cox-proportional hazard models were constructed to assess the impact of co-variates; variables with *p* < 0·1 in univariable analyses were included in multivariable models. Differences in median values between groups were assessed using Wilcoxon rank sum test. Frequencies between groups were compared using Pearson’s χ^2^ test or Fisher’s exact test as appropriate. Confidence intervals around proportions were calculated using the Clopper-Pearson method. Data missingness is reported where this occurs with no imputation performed. For all tests, *p* values < 0·05 were considered statistically significant. Data were analysed using R (v4·3·2; packages in Supplementary Methods).

Reporting of this work has been performed in line with STROBE guidelines [24] and with the CONSIDER statement [25], with details of the governance and ethics of Hep B PAST described in detail elsewhere [17].

## Results

### Antigen loss in sub-genotype C4 chronic hepatitis B infection

**Fig. 1:**
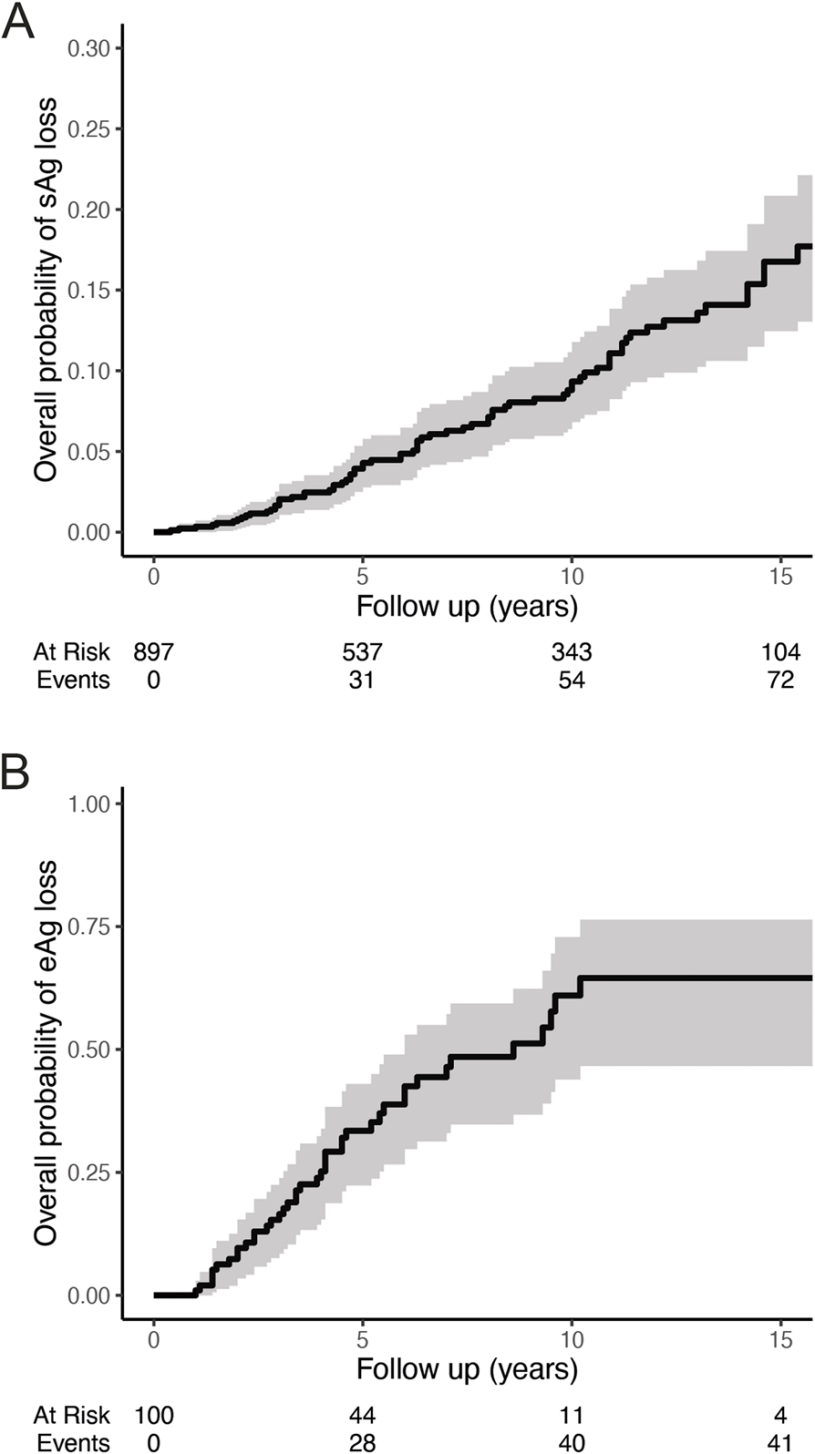
Loss of hepatitis B antigens. Kaplan–Meier plots showing (**A**) loss of HBsAg and (**B**) loss of HBeAg over first 15 years of follow up. 95% confidence intervals are shaded in grey

The rate of HBsAg loss was assessed amongst 1061 individuals identified with chronic hepatitis B of whom 897 had sufficient data for inclusion in analysis (exclusions shown in Supplementary Fig. 5). Follow up was median 7·1 years [IQR 2·8–12·1] with median age of 39 years [IQR 31–51] at first HBsAg. Loss of HBsAg occurred in 76 individuals over a follow up of 7342·6 person years, giving a rate of 1·04 events/100 person-years of follow up. The probability of remaining HBsAg positive was 91% at ten years [95% CI 88–93] (Fig. 1A). Loss of HBsAg was independently associated with male sex (HR 2·01 [1·22–3·33]) and age (HR 1·04 [1·03–1·06], overall *p* < 0·001). HBsAg loss was associated with HBeAg status at univariable level but did not persist after adjustment for age and sex (Supplementary Table 1).

HBeAg loss was quantified among 100 individuals, after assessment of 1061 for potential inclusion (exclusions shown in Supplementary Fig. 6). Follow up was median 4·5 years [IQR, 2·5–7·5] with median age of 31 years [IQR 24–37] at first HBeAg result. HBeAg loss occurred in 44 individuals over 545·6 person years of follow up, equating to 8·06 events/100 person-years of follow up and a probability of remaining HBeAg positive of 39% at ten years [95% CI 27–56] (Fig. 1B). Loss of HBeAg was associated with male sex (HR 2·09 [1·09–4·04], *p* = 0·028). HBeAg loss was also associated with age in univariable analysis (HR 1·03 [1·00–1·07], *p* = 0·048) but this was no longer significant after adjustment for sex (Supplementary Table 2). In this cohort, 17 individuals (2% of those with serial HBeAg measures) had a positive HBeAg which followed a previous negative antigen, including nine who were observed to lose and regain.

### Changes in disease phase, and progression to treatment in sub-genotype C4 chronic hepatitis B infection

**Fig. 2:**
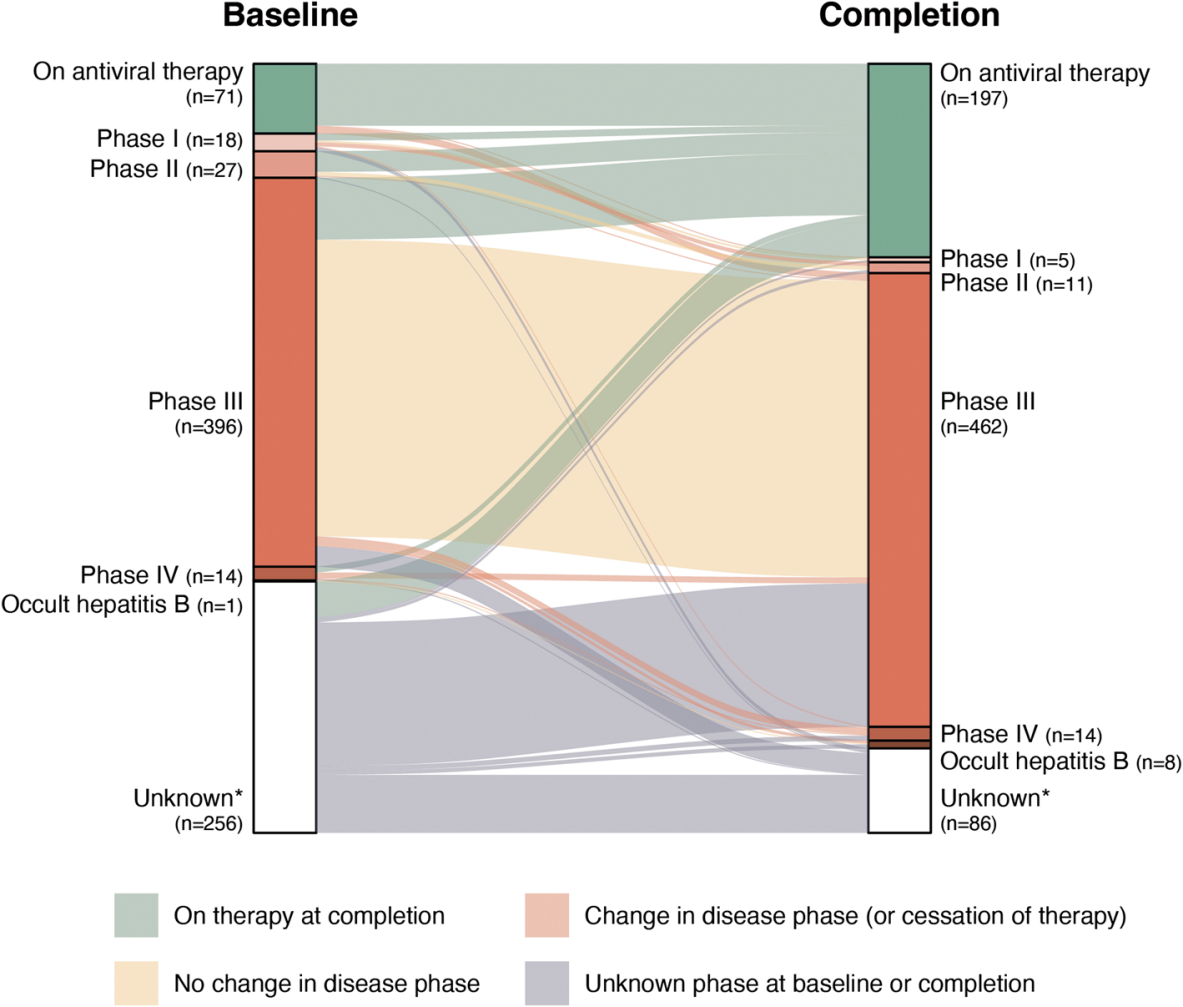
Changes in disease phase and treatment status. Alluvial plot showing comparison of number of individuals by treatment/disease status at baseline and study completion. Data flows represent the changes in status between time points and are proportional to the number of individuals. Individuals are classified as unknown if insufficient information was available to assign disease phase. Alternate names for phases as follows: phase I (HBeAg positive chronic infection, or immune tolerant), phase II (HBeAg positive chronic hepatitis or immune clearance), phase III (HBeAg negative chronic infection or immune control) and phase IV (HBeAg negative chronic hepatitis or immune escape). Data used in construction of figure is shown in Supplementary Table 3

At completion of Hep B PAST, 821 individuals were listed as having chronic hepatitis B. Following manual case review of those with negative HBsAg (exclusions shown in Supplementary Fig. 7), 783 individuals were included in subsequent analyses. A disease phase was assigned to these individuals who were untreated at both analysis time points (712 at baseline and 586 at completion). Sufficient data was available to assign a disease phase in 64% at baseline and 85% at completion. Disease phase and treatment status at both timepoints are represented visually in Fig. 2, where flows represent the changes in status between time points and are proportional to the number of individuals.

Between baseline and completion, 134 individuals were commenced on therapy. These include most individuals known to be in phase II or IV at baseline (78% and 43% of those groups respectively). 332 individuals off treatment at both time points had sufficient data to have a disease phase assigned. The majority (93%) remained in the same disease phase, with movement between phases only observed in 22 (7%) (Fig. 2, Supplementary Table 3).

### Clinical status of sub-genotype C4 chronic hepatitis B infection at the completion of Hep B PAST

The majority (60%) of individuals with CHB were male and the overall cohort had a median age of 48 years (IQR 40–58) at completion. 16% have established cirrhosis, with an additional 6% having FibroScan > 7 kPa (total 22% cirrhosis or significant fibrosis). Characteristics of all individuals at completion (disaggregated by sex) are shown in Table 1. 25% of individuals with CHB were treated with antiviral therapy (Fig. 2). Individuals on therapy were more likely than untreated individuals to have clinical features which form part of treatment indications: cirrhosis (39% versus 6·7%, *p* < 0·001), a higher baseline ALT (median 28 versus 23 IU/L, *p* < 0·001) and HBeAg positivity (23% versus 3·5%, *p* < 0·001) (Supplementary Table 4).

**Table 1:** Characteristics of people living with chronic hepatitis B in the Northern Territory by sex

Sex	n	Female	Male	*p*-value
Number of individuals	783	310	473	
Treated with antivirals	783			0.2
- Yes		85 (27%)	112 (24%)	
- No		225 (73%)	361 (76%)	
Age (years)	783	49 (41, 59)	47 (39, 57)	0.046
Remoteness	783			0.7
- Very remote		307 (99%)	466 (99%)	
- Remote		3 (1.0%)	7 (1.5%)	
HBeAg	716			0.2
- Positive		28 (10%)	32 (7.3%)	
- Negative		250 (90%)	406 (93%)	
HBeAb	716			0.8
- Positive		227 (82%)	357 (82%)	
- Negative		51 (18%)	79 (18%)	
- Equivocal		0	2 (0.5%)	
Median liver stiffness score (kPa)	423	5.1 (4.1, 7.6)	5.2 (4.2, 6.7)	> 0.9
Liver stiffness score	423			
- ≤ 7 kPa		123 (72%)	196 (78%)	0.3
- 7–10 kPa		18 (10%)	23 (9.2%)	
- > 10 kPa		31 (18%)	32 (13%)	
APRI	415			0.7
- ≤ 0.5		157 (91%)	215 (89%)	
Done- 0.5–2		14 (8.1%)	25 (10%)	
Done- > 2		2 (1.2%)	2 (0.8%)	
Cirrhosis status	609			0.7
Done- No cirrhosis		206 (83%)	304 (84%)	
Done- Cirrhosis		42 (17%)	57 (16%)	
ALT (IU/L)	710	20 (16, 29)	27 (19, 36)	< 0.001

Clinical variables are shown by sex at completion of study follow up. Numbers are shown as n (%) for categorical variables and median (interquartile range) for continuous variables. Groups have been compared with Pearson’s chi-squared test or Fisher’s exact test (categorical) or Wilcoxon rank sum test (continuous variables)

*Abbreviations used: APRI* aspartate aminotransferase to platelet ratio, alanine aminotransferase

### Assessment of treatment need against current local guidelines, and expanded international treatment criteria

Hep B PAST has been shown to improve the cascade of care for hepatitis B among First Nations people in the NT [17]. In line with this, the proportion of individuals enrolled in Hep B PAST who were prescribed antivirals was higher at completion than baseline (12% to 25%, *p* < 0·001; Supplementary Table 5). Additionally, the proportion of individuals with sufficient information to assess treatment need against Australian guidelines increased from 73 to 89% (*p* < 0·001; Supplementary Table 6).

For individuals not currently on antiviral therapy, the need for therapy was assessed based on Australian (GESA) guidelines [2] which consider the presence of cirrhosis, disease phase, ALT and HBV DNA (Supplementary Fig. 2). At completion, 500 untreated individuals (85% of 586 not on therapy) had sufficient data to assess treatment need of whom 39 (6·7%) met the criteria for treatment initiation (Table 2).

**Table 2:** Indications for antiviral therapy amongst untreated people living with chronic hepatitis B

Guideline used	GESA 2022 (2)	EASL 2017 (4)	WHO 2024 (6) (limited application)	WHO 2024 (6) (considering diabetes)
Antivirals indicated	39 (6.7%)	42 (7.2%)	91 (16%)	294^a^ (50%)
Antivirals not indicated	461 (79%)	458 (78%)	439 (75%)	-
Insufficient data to assess	86 (15%)	86 (15%)	56 (9.6%)	-

Indication for antiviral therapy amongst 586 untreated people living with chronic hepatitis B at completion of Hep B PAST as assessed against Gastroenterological Society of Australia (GESA) guidelines(2), European Association for the Study of the Liver (EASL)(4) and World Health Organisation (WHO) guidelines(6). Limited application of WHO guidelines considered only fibrosis/cirrhosis status (APRI > 0·5 or TE showing median stiffness > 7 kPa) or HBV DNA > 2000 copies/mL with raised ALT as indications for therapy. Age and sex-specific prevalence of diabetes was applied to provide more complete application of WHO guidelines

^a^Includes an estimated 203 individuals [95% confidence interval 181–225] who are eligible for treatment because of diabetes mellitus without other indications

The EASL guidelines broadly align with Australian guidelines on the indications for treatment but additionally have provision to treat if HBeAg positivity persists to > 30 years, or with moderate fibrosis with raised HBV DNA [4]. Using these, an additional three untreated individuals met the criteria for treatment initiation (total 42, 7·2%, Table 2); all were HBeAg positive and aged > 30 years.

The 2024 WHO guidelines for chronic hepatitis B simplify and expand the indications for therapy. Treatment in the WHO guidelines is indicated in the setting of significant fibrosis or cirrhosis (APRI > 0·5 or TE showing median stiffness > 7 kPa) or HBV DNA > 2000 copies/mL with ALT above sex-specific cut-offs [6]. Applying this criterion only (limited guideline application), 530 untreated individuals (90% of 586 not on therapy) had sufficient data to assess treatment need of whom 91 met the criteria for treatment initiation (16%, Table 2); for 76 of these this was due to evidence of fibrosis alone (Supplementary Table 7). The WHO guidelines also consider the presence of co-infections, family history of HCC or cirrhosis, immune suppression, extrahepatic manifestations of HBV or comorbidities (such as diabetes or metabolic dysfunction-associated steatotic liver disease [MASLD]) as indications for antiviral therapy [6]; these clinical variables are not present in our data. Noting the high prevalence of diabetes in our setting (29% of adults in recent estimates [20]), we anticipate these clinical criteria will substantially further expand those for whom antiviral therapy is indicated. We applied published age and sex-specific estimates of diabetes prevalence in First Nations people from remote communities in the NT from 2018–2019 [20] to 495 individuals in whom antiviral therapy was not indicated based on other criteria, or had insufficient information to assess. Doing so, we estimate an additional 203 individuals [95% CI 181–225 individuals] would meet the criteria for antiviral therapy, increasing the treatment gap to 50% (Table 2). Considering those already on therapy, this would mean that antiviral treatment is indicated in at least 63% of those living with CHB in our setting.

## Discussion

We report on the largest cohort of individuals with sub-genotype C4 hepatitis B infection. The majority of First Nations people in the NT live in remote communities [26] spread across a vast geographic area. The inclusion of real-world data from 76% of remote community clinics in the NT [17] is a key strength of this study and provides a representative snapshot of C4 sub-genotype infection. Here we observed that 22% of the cohort have evidence of cirrhosis (based on a composite of clinical documentation, FibroScan and APRI) or significant fibrosis (FibroScan > 7 kPa). This is consistent with our prior report of cirrhosis amongst 13% of those living with sub-genotype C4 infection [16], and supports that our previous estimate is not a result of bias through identification of those with more advanced disease. Whilst we observe a severe phenotype of liver disease amongst those living with C4 infection, co-existent risk factors for liver disease (such as cohort age and diabetes) make it difficult to know to what extent the viral sub-genotype itself is responsible for these severe outcomes.

We can probe the possibility of genotype-specific effects by comparing rates of loss of HBsAg and HBeAg in C4 infection with estimates from other genotypes. We observed loss of HBsAg at approximately 1% per year; loss of HBeAg occurred at approximately 8% per year. These are consistent with large meta-analyses of published estimates from mixed hepatitis B genotypes [27, 28]. Age and HBeAg are linked, with lower seroprevalence in older age groups [29, 30] and prolonged HBeAg positivity, a marker of viral replication, is associated with the development of cirrhosis [31] and HCC [32]. Longitudinal studies show that compared with other genotypes, HBeAg clearance is delayed in genotype C infection [21, 33–35]. HBeAg prevalence in our study (8·4% of participants, median age 48 years) is lower than previously reported age-specific estimates of HBeAg seropositivity amongst individuals aged > 40 across multiple regions globally [30] and when considering genotype C alone [29]. Overall, our estimates of antigen loss in sub-genotype C4 infection are similar to those from other genotypes. These findings support a need to study how other factors might contribute to the advanced phenotype observed in our population of people living with CHB. To understand the impact of genotype on development of HCC and progressive fibrosis, prospective data is needed. Follow up of the CHARM study [16], which is prospectively documenting clinical outcomes and circulating genotypes of HBV in First Nations people throughout Australia, and the Hep B PAST cohort is ongoing and will provide these answers for sub-genotype C4.

Supporting the success of the Hep B PAST Program [17] under current Australian treatment guidelines [2] we report a small treatment gap, with only 6·7% of those not on antiviral therapy qualifying for treatment. With broadened treatment indications in the 2024 WHO Guidelines [6] there is a substantial increase in those eligible for treatment to > 60% of those living with CHB. In our cohort this has been driven by high rates of fibrosis and population prevalence of diabetes. We were not able to fully apply the WHO treatment criteria and unmeasured treatment indications would be expected to increase this further, albeit not substantially. Hepatitis D virus infection has not been observed with sub-genotype C4 infection [36] and rates of HIV and HCV co-infection are low in local seroprevalence studies of First Nations people [37, 38]. The frequency of MASLD in our population is unknown and an area for future study. Diabetes is a key risk factor for MASLD [39] and in Queensland (also in northern Australia) First Nations people are diagnosed with MASLD at younger ages than non-Indigenous Australians [40]; these demographic features suggest the prevalence of MASLD in our population is likely to be high. End-stage renal failure is prevalent in our population [21], with diabetes the main biopsy-proven cause [41]; the contribution of hepatitis B infection to rates of glomerulonephritis is unclear [41, 42]. Overall, we anticipate the inclusion of diabetes in our analysis will capture most of those with additional treatment indications.

Under updated global guidelines, we show that a substantial proportion of currently untreated individuals with CHB have indications for antiviral therapy due to significant fibrosis and prevalence of diabetes. The use of nucleos(t)ide analogues for those infected with CHB is a safe and simple intervention which may have significant impact on preventing poor outcomes. Even if the liver disease in our population is multifactorial, addressing all reversible risk factors for progressive liver disease and HCC development is a pragmatic approach; the inclusion of these comorbidities as an indication for such therapy in WHO guidelines is a recognition of this. The provision of antiviral therapy alongside other interventions for liver health (such as alcohol reduction interventions and addressing diabetes control) should be done alongside further research to understand the impact of these interventions on progressive fibrosis and HCC development.

### Limitations

Identifying individuals for inclusion using an EHR serostatus, which was systematically placed for all individuals attending each clinic, protects against some selection biases but the cohort design has other risks, including that of unmeasured confounders. We acknowledge that collection of data as part of routine care means that has led to some data missingness and have reported where this occurs. An additional limitation of the study is that treatment status was only recorded in our cohort at two points, and we were therefore unable to draw conclusions about the impact of therapy.

## Conclusions

We show that significant fibrosis and cirrhosis are highly prevalent in a population infected with sub-genotype C4 hepatitis B. It is unclear to what extent this fibrosis is driven by sub-genotype C4 virus itself, versus comorbidities. Indeed, our results demonstrate that rates of HBsAg loss, HBeAg loss (and prevalence) appear to align with estimates from other genotypes. This supports the need for further studies to understand the development of fibrosis in this group including the contribution of co-existent MASLD and diabetes mellitus. Clinical data about First Nations people living with chronic hepatitis B are essential to support context-specific decisions to expand access to antiviral therapy and strengthen health outcomes.

## Supplementary Information

Supplementary Material 1.

## Data Availability

Ethical and privacy considerations restrict public access to the data collected and analysed in this study. Data will not be shared prior to the final analysis being published by the study’s authors. Following this time, data access proposals will require consideration and approval by the study management committee (the data custodians). Data requests can be made through the Hep B PAST steering committee, email: Hepbpast@menzies.edu.au.

## Abbreviations

ALT: Alanine aminotransferase
APRI: AST to platelet ratio index
HBV: Hepatitis B virus
HCV: Hepatitis C virus
CHB: Chronic hepatitis B
EASL: European Association for the Study of the Liver
EHR: Electronic health record
GESA: Gastroenterological Society of Australia
HCC: Hepatocellular carcinoma
HRN: Hospital record number
MASLD: Metabolic dysfunction-associated steatotic liver disease
NT: Northern Territory
TE: Transient elastography
WHO: World Health Organisation
