# Supplementary material for: Disease progression & treatment need in sub-genotype C4 hepatitis B infection: a retrospective cohort study in the Northern Territory, Australia
Source: BMC Infect Dis. 2025 Jul 1;25:881. doi: 10.1186/s12879-025-11213-w (PMC12219930; doi:10.1186/s12879-025-11213-w)
Supplement: Supplementary file 1 — Supplementary Material 1. [file 12879_2025_11213_MOESM1_ESM.pdf]

Disease progression & treatment need in sub-genotype C4 hepatitis B infection: a retrospective cohort study in the Northern Territory, Australia

Genevieve E Martin, Kelly Hosking, Kelly Banz, Catherine Gargan, Geoff Stewart, Belinda Greenwood-Smith, Penelope Ramsay, Jaclyn Tate-Baker, Christine Connors, Paula Binks, Melita McKinnon, Prashanti Manchikanti, George Garambaka Gurruwiwi, Nicole Allard, Ashleigh Qama, Jessica Michaels, Emily Vintour-Cesar, Robert Batey, Catherine Marshall, Peter Nihill, Tammy-Allyn Fernandes, Karen Fuller, Steven YC Tong, David Boettiger, Benjamin Cowie, Joshua S Davis, Sarah Mariyalawuy Bukulatjpi, Jane Davies on behalf of the Hep B PAST Partnership.

SUPPLEMENTARY DATA

Contents

Hep B PAST Partnership Members and Individuals 2

Supplementary Methods 6

Supplementary Table 1. Cox-proportional hazards models for assessing factors associated with HBsAg loss 8

Supplementary Table 2. Cox-proportional hazards models for assessing factors associated with HBeAg loss 8

Supplementary Table 3. Changes in disease phase and treatment status 9

Supplementary Table 4. Characteristics of people living with chronic hepatitis B in the Northern Territory by treatment status 10

Supplementary Table 5. Change in proportion of individuals on antiviral therapy between time points 11

Supplementary Table 6. Change in proportion of individuals with sufficient data to assess treatment need between time points 11

Supplementary Table 7. Indications for antiviral therapy using assessment of 2024 World Health Organisation guidelines 11

Supplementary Figure 1. Logic used for assignment of hepatitis B disease phase 12

Supplementary Figure 2. Logic used to assess need for antiviral therapy against Gastroenterological Society of Australia guidelines 13

Supplementary Figure 3. Logic used to assess need for antiviral therapy against European Association for the Study of the Liver guidelines 14

Supplementary Figure 4. Logic used to assess need for antiviral therapy against World Health Organisation guidelines 15

Supplementary Figure 5. Individuals included in assessment of HBsAg loss 16

Supplementary Figure 6. Individuals included in assessment of HBeAg loss 16

Supplementary Figure 7. Individuals included in assessment of hepatitis B disease phase and treatment need ... 17

Supplementary References 18

Hep B PAST Partnership Members and Individuals

Partner Organisations

Menzies School of Health Research
Northern Territory Government, NT Health
ASHM
Katherine West Health Board Aboriginal Corporation
Miwatj Health Aboriginal Corporation
NT AIDS and Hepatitis Council, NTAHC
Mala’la Health Service Aboriginal Corporation
Marthakal Homelands Health Service
Central Australian Aboriginal Congress

Associate Partner Organisations

WHO Collaborating Centre for Viral Hepatitis, VIDRL, Doherty Institute
Victorian Infectious Diseases Reference Laboratory - VIDRL
Hepatitis Australia

Investigators and Contributors

Jane Davies	Menzies School of Health Research and NT Health	CI
Sarah Mariyalawuy Bukulatjpi	Miwatj Health Aboriginal Corporation	CI
Christine Connors	NT Health	CI
Joshua Davis	Menzies School of Health Research	CI
Robert Batey	NT Health	CI
Benjamin Cowie	WHO Collaborating Centre for Viral Hepatitis, VIDRL, Doherty Institute	CI
Steven Tong	Doherty Institute for Infection and Immunity	CI
Anna Ralph	Menzies School of Health Research	CI
Adrian Miller	Central Queensland University	CI
Kelly Hosking	NT Health and Menzies School of Health Research	AI
George Garambaka Gurruwiwi	Menzies School of Health Research	AI
Roslyn Dhurrkay	Miwatj Health Aboriginal Corporation	AI
Belinda Greenwood-Smith	NT Health	AI
Catherine Marshall	NT Health	AI
Geoffrey Stewart	NT Health	AI
Nicole Allard	WHO Collaborating Centre for Viral Hepatitis, VIDRL, Doherty Institute	AI
Manoji Gunthilake	NT Health	AI
Vicki Krause	NT Health	AI
Ashleigh Qama	WHO Collaborating Centre for Viral Hepatitis, VIDRL, Doherty Institute	PI
Karen Fuller	Katherine West Health Board Aboriginal Corporation	PI
Jessica Michaels	ASHM	PI
Lou Sanderson	Miwatj Health Aboriginal Corporation	PI
Phillip Merrdi Wilson	NT Health	PI
Genevieve Dally	NTAHC	PI
Kerrie Jordan	NTAHC	PI

John Boffa	Central Australian Aboriginal Congress	PI
Alexis Apostolellis	ASHM	P
Amanda Dhagapan	Miwatj Health Aboriginal Corporation	P
Anna Deng	WHO Collaborating Centre for Viral Hepatitis, VIDRL, Doherty Institute	P
Anngie Everitt	Menzies School of Health Research	P
Barbara De Graaff	Menzies Institute for Medical Research	P
Brianna Summers	Katherine West Health Board Aboriginal Corporation	P
Carrie Fowler	Hepatitis Australia	P
Catherine Blacker	NT Health and Menzies School of Health Research	P
Catherine Gargan	NT Health	P
Catherine Stoddart	NT Health	P
Charles Pain	NT Health	P
Cheryl Ross	Menzies School of Health Research	P
David McGuinness	Katherine West Health Board Aboriginal Corporation	P
David Reeve	NT Health	P
Diane Hampton	Katherine West Health Board Aboriginal Corporation	P
Eddie Mulholland	Miwatj Health Aboriginal Corporation	P
Ella Meumann	NT Health	P
Elizabeth Coombes	NT Health	
Emily Vintour-Cesar	Menzies School of Health Research	P
Emma Childs	NT Health and Miwatj Health Aboriginal Corporation	P
Hayden Jose	ASHM	P
Hilary Bloomfield	Miwatj Health Aboriginal Corporation	P
Hugh Heggie	NT Health	P
Isabelle Purcell	ASHM	P
Jaclyn Tate-Baker	NT Health	P
Jayne Porter	NT Health	P
Jyoti Jadeja	NTAHC	P
Katherine McNamara	NT Health	P
Katie McGuire	Menzies School of Health Research	P
Keith Forrest	Mala'la Health Service Aboriginal Corporation	P
Kelly Banz	NT Health	P
Kelly-Anne Stuart-Carter	NT Health	P
Khim Tan	NT Health	P
Leanne O'Connor	NT Health	P
Lesley Scott	NT Health	
Letisha Murray	NT Health	P
Levinia Crooks^	ASHM	P
Linda Bunn	NT Health	P

Lorraine Johns	Katherine West Health Board Aboriginal Corporation	P
Lucie Perrisel	ASHM	P
Marco Briceno	NT Health	P
Margaret Littlejohn	Doherty Institute for Infection and Immunity	P
Maria Scarlett	NTAHC	P
Marilou Capati	Top End Medical Centre	P
Matthew Maddison	NT Health	P
Melita McKinnon	Menzies School of Health Research	P
Mikaela Mobsby	NT Health	P
Molly Shorthouse	Miwatj Health Aboriginal Corporation	P
Natasha Tatipata	NT Health	P
Nicole Romero	WHO Collaborating Centre for Viral Hepatitis, VIDRL, Doherty Institute	P
Paula Binks	Menzies School of Health Research	P
Penny Ramsey	NT Health	P
Peter Nihill	NT Health	P
Peter Markey	NT Health	P
Phoebe Schroder	ASHM	P
Prashanti Manchikanti	Miwatj Health Aboriginal Corporation	P
Rebecca Katiforis	NT Health	P
Robyn Liddle	Menzies School of Health Research	P
Rosalind Webby	NT Health	P
Richard Sullivan	Menzies School of Health Research	P
Sami Stewart	ASHM	P
Sandra Nelson	NT Health	P
Sean Heffernan	Katherine West Health Board Aboriginal Corporation	P
Sean Taylor	NT Health	P
Shiraline Wurrawilya	NT Health	P
Sinon Cooney	Katherine West Health Board Aboriginal Corporation	P
Sonja Hill	ASHM	P
Stephen Locarnini	Doherty Institute for Infection and Immunity	P
Steven Skov	NT Health	P
Su Govindasamy	ASHM	P
Sudharsan Venkatesan	Menzies School of Health Research and NT Health	P
Tammy-Allyn Fernandes	NT Health	P
Tanya Plavins	Central Australian Aboriginal Congress	P
Teresa De Santis	NT Health	P
Terese Ngurruwuthun	Miwatj Health Aboriginal Corporation	P
Tiana Alley	Menzies School of Health Research	P
Timothy Nabegeyo	NT Health	P

Vanessa Towell	ASHM	P
Wendy Page	Miwatj Health Aboriginal Corporation	P

Key: CI - Chief Investigator, AI- Associate Investigator, PI – Partner Investigator, P – Partner, ^ - Deceased

Supplementary Methods

Clinical variables:

Treatment status (if patient was prescribed nucleos(t)ide analogues or not) was contained as part of the HBV status and was only available at two time points: October 2020 (initial allocation of hepatitis B status and the creation of the Hep B Hub) and the completion of the program (October 2023); the exact date of treatment initiation was not available. HBV DNA was only used to assess treatment need in individuals not on therapy. Values are not reported across the cohort as these are not interpretable without more precise data about timing of measurement relative to treatment initiation (as complete viral load suppression on nucleos(t)ide analogue therapy can take several years(1)), and direct assessment of prescription, dispensing and adherence to antivirals.

Data inclusion:

Values prior to the three-year window were included only in the following scenarios where it would be clinically appropriate for these to not be repeated: HbeAb and HbeAg (if HBeAb positive and HBeAg negative), and transient elastography (TE) with FibroScan (Echosens, Paris, France) (if previous FibroScan >10 kPa). Cirrhosis was defined as the presence of any of a) clinical/radiographic evidence of cirrhosis, b) FibroScan >10 kPa (a cutoff previously used in C4 sub-genotype infection(2)) or c) AST to platelet ratio (APRI) >2. An individual was only designated as not having cirrhosis if FibroScan or APRI was available and below cutoff in the three years prior to analysis time point.

Determination of disease phase:

Individuals were allocated a disease phase based on Gastroenterological Society of Australia (GESA) guidelines (3) Where possible, this was determined algorithmically (as shown in Supplementary Figure 1). Manual case review of all available information was performed to assign a phase where HBsAg was negative, both HBeAg and HBeAb were negative (or equivocal), or HBeAg was positive in the setting of HBV DNA <20000 IU/mL

Software used for data analysis:

Data were analysed using R (v4·3·2) using packages tidyverse (v2·0·0), survival (v3·5-7) and PropCIs (v0·3-0)). The packages ggsurvfit (v1·0·0), survminer (v0·4·9) and ggalluvial (v0·12·5) were used in the creation of figures.

Supplementary Table 1. Cox-proportional hazards models for assessing factors associated with HBsAg loss

	Univariable			Multivariable		
	n	***p***	**HR [95% CI]**	***n***	***p***	**HR [95% CI]**
Sex	897	0·008	1·99 [1·20 – 3·30]	897	0·007	2·01 [1·22 – 3·33]
HBeAg status	811	0·022	0·10 [0·01 – 0·72]		-	-
Age at first HBsAg	897	<0·001	1·04 [1·03 – 1·06]		<0·001	1·04 [1·03 – 1·06]

Cox proportional hazards models to assess associations with HBsAg loss per year. n = 897 with 61 individuals excluded from analyses containing HBeAg status due to missingness. For sex the reference group was male, and for HBeAg status the reference group was positive. Variables significantly associated with the outcome (at $p<0·1$) were included in the multivariable model with stepwise elimination where $p>0·05$. For multivariable model, overall $p < 0·001$ via logrank test.

Abbreviations: HR, hazard ratio; CI, confidence interval.

Supplementary Table 2. Cox-proportional hazards models for assessing factors associated with HBeAg loss

	Univariable			Multivariable		
	n	***p***	**HR [95% CI]**	***n***	***p***	**HR [95% CI]**
Sex	100	0·028	2·09 [1·09 - 4·04]	100	0·041	2·00 [1·03 – 3·83]
Age at first HBeAg	100	0·048	1·03 [1·00 – 1·07]	100	0·074	1·03 [1·00 – 1·06]

Cox proportional hazards models to assess associations with HBeAg loss per year, n = 100. For sex the reference group was male. For multivariable model, overall $p = 0·02$ via logrank test.

Abbreviations: HR, hazard ratio; CI, confidence interval.

Supplementary Table 3. Changes in disease phase and treatment status

Baseline	Completion	n
On antiviral therapy	On antiviral therapy	63
Phase I	On antiviral therapy	7
Phase II	On antiviral therapy	21
Phase III	On antiviral therapy	63
Phase IV	On antiviral therapy	6
Unknown	On antiviral therapy	37
On antiviral therapy	Phase I	1
Phase I	Phase I	2
Unknown	Phase I	2
Phase I	Phase II	4
Phase II	Phase II	4
Unknown	Phase II	3
On antiviral therapy	Phase III	7
Phase II	Phase III	1
Phase III	Phase III	302
Phase IV	Phase III	6
Unknown	Phase III	146
Phase I	Phase IV	1
Phase III	Phase IV	7
Phase IV	Phase IV	1
Unknown	Phase IV	5
Phase III	Occult hepatitis B	3
Occult hepatitis B	Occult hepatitis B	1
Unknown	Occult hepatitis B	4
Phase I	Unknown	4
Phase II	Unknown	1
Phase III	Unknown	21
Phase IV	Unknown	1
Unknown	Unknown	59

Comparison of number of individuals by treatment/disease status at baseline and completion. Data in this table is represented visually in Figure 2. Individuals are classified as unknown if insufficient information was available to assign disease phase algorithmically or after manual review as shown in Supplementary Figure 1. Alternate names for phases as follows: phase I (HBeAg positive chronic infection, or immune tolerant), phase II (HBeAg positive chronic hepatitis or immune clearance), phase III (HBeAg negative chronic infection or immune control) and phase IV (HBeAg negative chronic hepatitis or immune escape).

Supplementary Table 4. Characteristics of people living with chronic hepatitis B in the Northern Territory by treatment status

Treated with antivirals	n	No	Yes	p-value
Number of individuals	783	586	197	
Sex	783			0·2
- Female		225 (38%)	85 (43%)	
- Male		361 (62%)	112 (57%)	
Age (years)	783	48 (40, 57)	49 (41, 59)	0·4
Remoteness	783			0·7
- Very remote		579 (99%)	194 (98%)	
- Remote		7 (1·2%)	3 (1·5%)	
HBeAg	716			<0·001
- Positive		19 (3·5%)	41 (23%)	
- Negative		518 (96%)	138 (77%)	
HBeAb	716			
- Positive		469 (87%)	115 (64%)	<0·001
- Negative		67 (12%)	63 (35%)	
- Equivocal		1 (0·2%)	1 (0·6%)	
Median liver stiffness score (kPa)	423	4·9 (4·0, 6·0)	6·5 (4·5, 12·1)	<0·001
Liver stiffness score	423			<0·001
- ≤7 kPa		237 (86%)	82 (55%)	
- 7 – 10 kPa		26 (9·5%)	15 (10%)	
- >10 kPa		12 (4·4%)	51 (34%)	
APRI	415			<0·001
- ≤0·5		285 (93%)	87 (80%)	
- 0·5 – 2		21 (6·9%)	18 (17%)	
- >2		0	4 (3·7%)	
Cirrhosis status	609			<0·001
- No cirrhosis		402 (93%)	108 (61%)	
- Cirrhosis		29 (6·7%)	70 (39%)	
ALT (IU/L)	710	23 (17, 33)	28 (20, 39)	<0·001

Clinical variables are shown by treatment status at completion of study follow up. Numbers are shown as n (%) for categorical variables and median (interquartile range) for continuous variables. Groups have been compared with Pearson's chi-squared test or Fisher's exact test (categorical) or Wilcoxon rank sum test (continuous variables). Abbreviations used: APRI, aspartate aminotransferase to platelet ratio; ALT, alanine aminotransferase.

Supplementary Table 5. Change in proportion of individuals on antiviral therapy between time points

Time point	Baseline	Completion	p-value
Number of individuals<br>Treated with antivirals	590	783	< 0·001
- Yes	71 (12%)	197 (25%)	
- No	519 (88%)	586 (75%)	

Contingency table showing number of individuals on antiviral therapy at both time points; numbers are shown as n (%). At baseline, 193 individuals in the cohort were not enrolled in Hep B PAST and did not have a treatment status recorded; these are excluded here. Groups compared using Pearson's chi-squared test.

Supplementary Table 6. Change in proportion of individuals with sufficient data to assess treatment need between time points

Time point	Baseline	Completion	p-value
Number of individuals<br>Sufficient data	590	783	< 0·001
- Yes	432 (73%)	697 (89%)	
- No	158 (27%)	86 (11%)	

Contingency table showing number of individuals with sufficient data to assess treatment need at both time points; numbers are shown as n (%). Individuals on treatment are considered to have sufficient data. At baseline, 193 individuals in the cohort were not enrolled in Hep B PAST and did not have a treatment status recorded; these are excluded here. Groups compared using Pearson's chi-squared test.

Supplementary Table 7. Indications for antiviral therapy using assessment of 2024 World Health Organisation guidelines

Indication for antiviral therapy	n (%)
- Fibrotic criteria (APRI >0·5 or FibroScan >7 kPA or known cirrhosis)	76 (84%)
- Elevated ALT and HBV DNA > 2000 copies /mL	13 (14%)
- Both of the above	2 (2·2%)

Reasons that antiviral therapy is indicated for 91 people assessed as needing antivirals with limited application of World Health Organisation (WHO) guidelines (as shown in Table 2).(4) Limited application of WHO guidelines considered only fibrosis/cirrhosis status (APRI >0·5 or TE showing median stiffness >7kPa) or HBV DNA >2000 copies/mL with raised ALT as indications for therapy.

Supplementary Figure 1. Logic used for assignment of hepatitis B disease phase

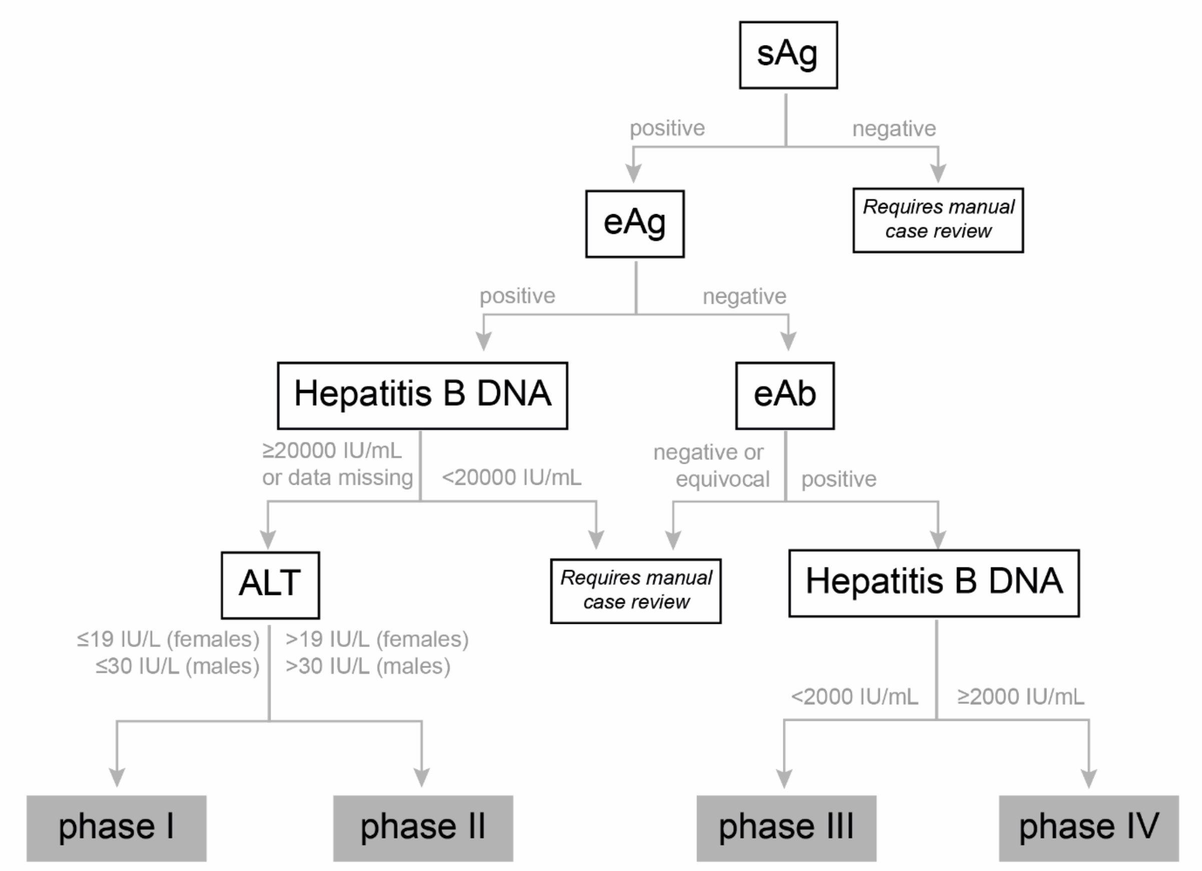

Schema is based on the Gastroenterological Society of Australia (GESA) guidelines.(3) Alternate names for phases as follows: phase I (HBeAg positive chronic infection, or immune tolerant), phase II (HBeAg positive chronic hepatitis or immune clearance), phase III (HBeAg negative chronic infection or immune control), phase IV (HBeAg negative chronic hepatitis or immune escape).Occult hepatitis B was defined as detectable hepatitis B DNA in absence of positive surface antigen and was only assigned after manual case review.

Supplementary Figure 2. Logic used to assess need for antiviral therapy against Gastroenterological Society of Australia guidelines

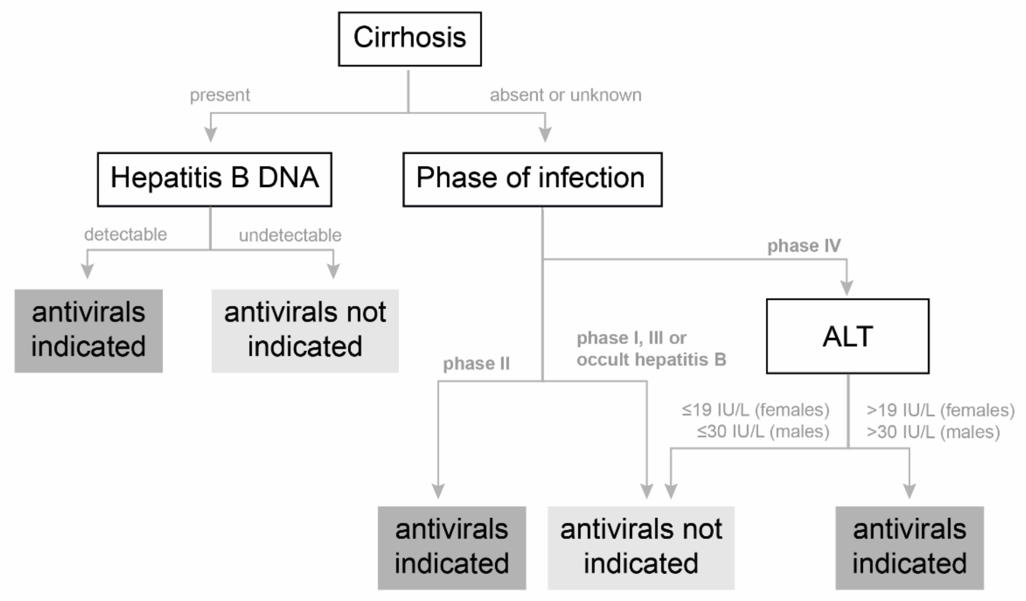

Schema is based on the Gastroenterological Society of Australia (GESA) guidelines.(3) For this assessment, an individual was considered to have a detectable HBV DNA if any measurement in the three-year window prior to assessment was detectable

Supplementary Figure 3. Logic used to assess need for antiviral therapy against European Association for the Study of the Liver guidelines

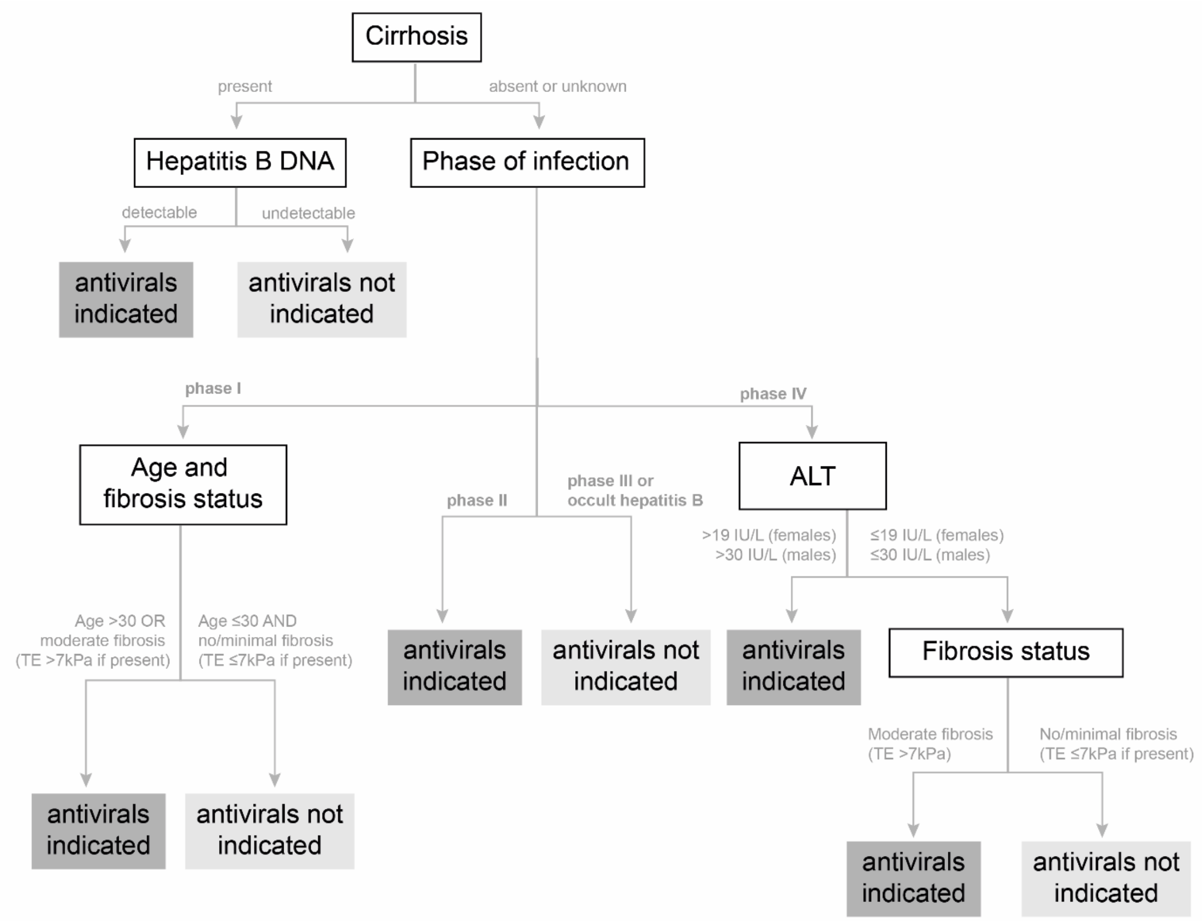

Schema is based on the European Association for the Study of the Liver (EASL) guidelines.(1) The EASLguidelines also consider a family history of HCC or cirrhosis or extrahepatic manifestations of HBV as indications for antiviral therapy; these clinical variables are not assessed in this schema. For this assessment, an individual was considered to have a detectable HBV DNA if any measurement in the three-year window prior to assessment was detectable

Supplementary Figure 4. Logic used to assess need for antiviral therapy against World Health Organisation guidelines

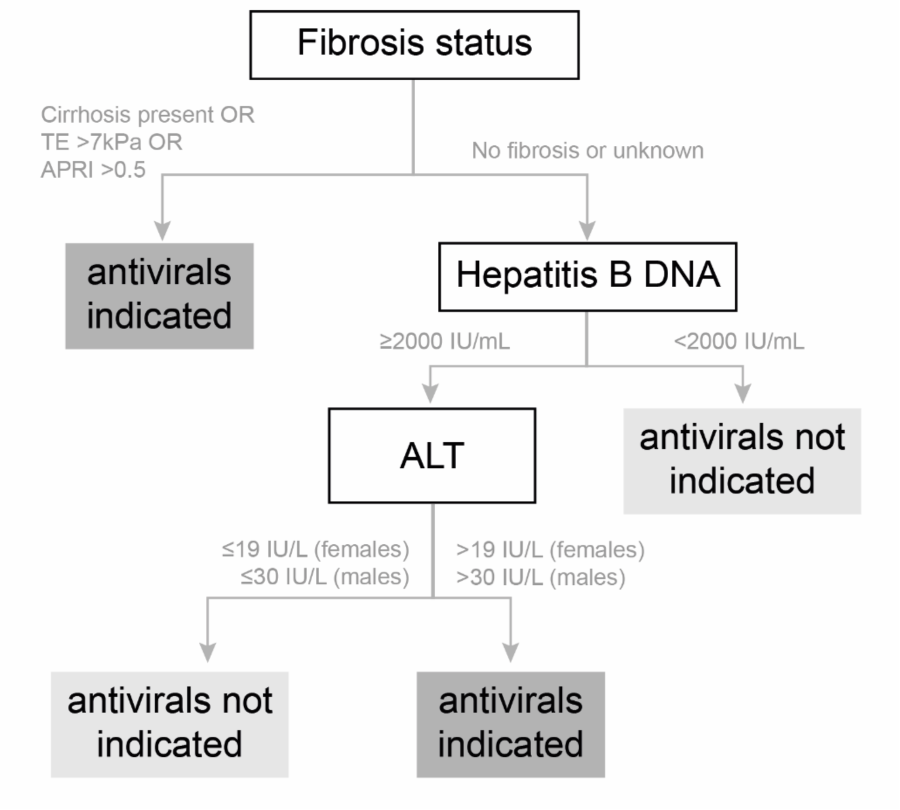

Schema is based on the European Association for the World Health Organisation (WHO) guidelines.(4) The WHO guidelines also consider the presence of co-infections, family history of HCC or cirrhosis, immune suppression, extrahepatic manifestations of HBV or comorbidities (such as diabetes or metabolic dysfunction-associated steatotic liver disease, MASLD) as indications for antiviral therapy; these clinical variables are not assessed in this schema.

Supplementary Figure 5. Individuals included in assessment of HBsAg loss

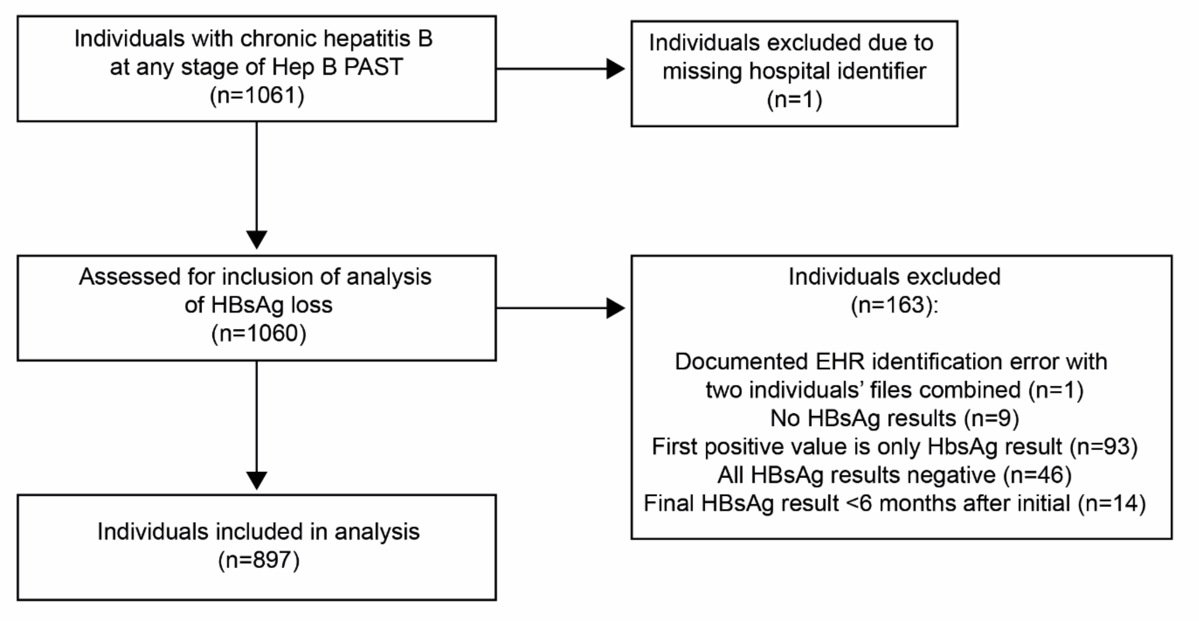

Supplementary Figure 6. Individuals included in assessment of HBeAg loss

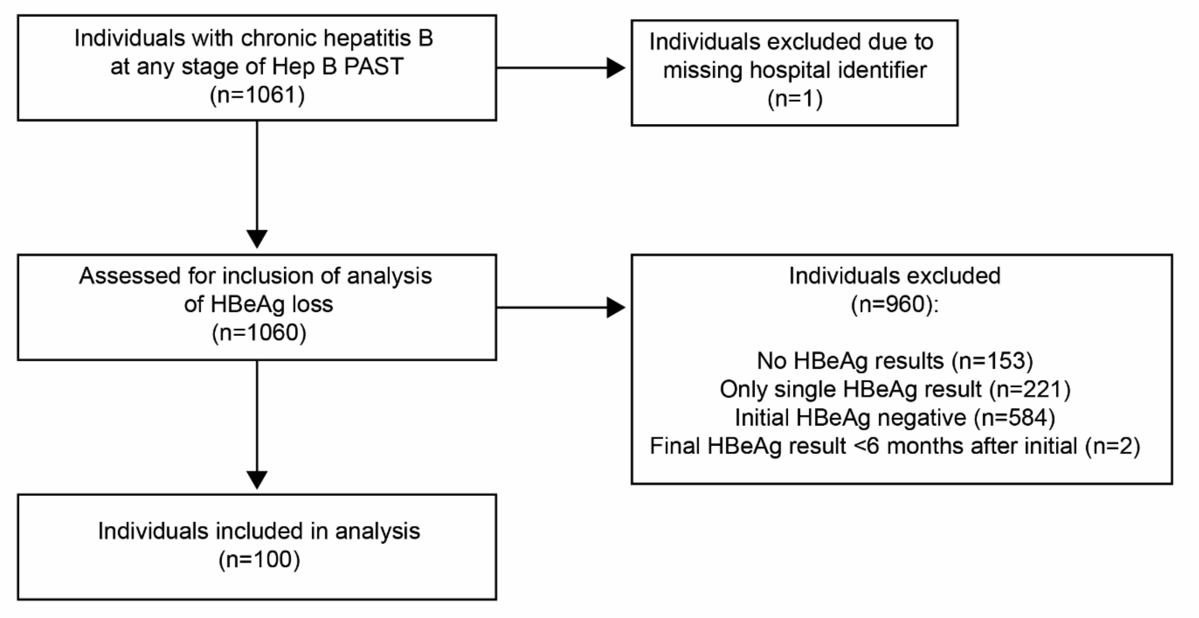

Supplementary Figure 7. Individuals included in assessment of hepatitis B disease phase and treatment need

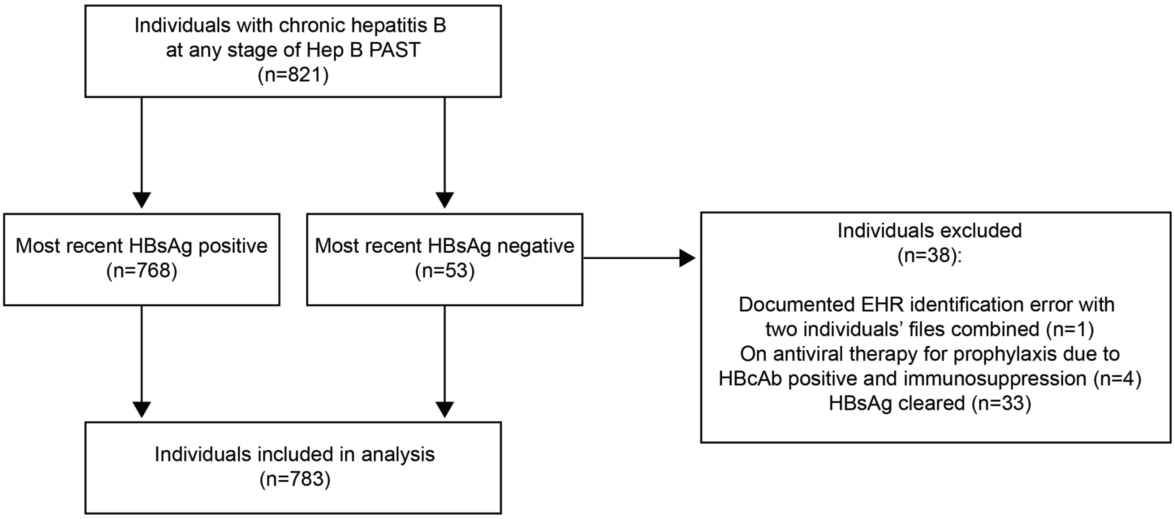

Manual case review of individuals whose most recent HBsAg was negative was performed and included serial HBsAg, HBV DNA and electronic records of clinician documentation. Individuals who had a negative HBsAg and were included in subsequent analysis had detectable HBV DNA (occult hepatitis B).

Supplementary References

1. European Association for the Study of the Liver. Electronic address eee, European Association for the Study of the L. EASL 2017 Clinical Practice Guidelines on the management of hepatitis B virus infection. J Hepatol. 2017;67(2):370-98.

2. Davies J, Smith EL, Littlejohn M, Edwards R, Sozzi V, Jackson K, et al. Towards Genotype-Specific Care for Chronic Hepatitis B: The First 6 Years Follow Up From the CHARM Cohort Study. Open Forum Infect Dis. 2019;6(11):ofz469.

3. Lubel JS, Strasser SI, Thompson AJ, Cowie BC, MacLachlan J, Allard NL, et al. Australian consensus recommendations for the management of hepatitis B. Med J Aust. 2022;216(9):478-86.

4. World Health Orgnisation. Guidelines for the prevention, diagnosis, care and treatment for people with chronic hepatitis B infection. Geneva: World Health Organisation; 2024. Contract No.: 978-92-4-009090-3.
